# Tranexamic acid is associated with reduced mortality, hemorrhagic expansion, and vascular occlusive events in traumatic brain injury – meta-analysis of randomized controlled trials

**DOI:** 10.1186/s12883-020-01694-4

**Published:** 2020-04-06

**Authors:** Julius July, Raymond Pranata

**Affiliations:** 1 Department of Neurosurgery, Medical Faculty of Pelita Harapan University, Lippo Village Tangerang, Neuroscience Centre Siloam Hospital, Lippo Village, Tangerang, Indonesia; 2 Faculty of Medicine, Universitas Pelita Harapan, Tangerang, Indonesia

**Keywords:** Brain trauma, Coagulopathy, Thromboembolism, Tranexamic acid, Traumatic brain injury, Vascular occlusive events

## Abstract

**Background:**

This systematic review and meta-analysis aimed to synthesize the latest evidence on the efficacy and safety of tranexamic acid (TXA) on traumatic brain injury (TBI).

**Methods:**

We performed a systematic literature search on topics that compared intravenous TXA to placebo in patients with TBI up until January 2020 from several electronic databases.

**Results:**

There were 30.522 patients from 7 studies. Meta-analysis showed that TXA was associated with reduced mortality (RR 0.92 [0.88, 0.97], *p* = 0.002; I^2^: 0%) and hemorrhagic expansion (RR 0.79 [0.64, 0.97], *p* = 0.03; I^2^: 0%). Both TXA and control group has a similar need for neurosurgical intervention (*p* = 0.87) and unfavourable Glasgow Outcome Scale (GOS) (*p* = 0.59). The rate for vascular occlusive events (*p* = 0.09), and its deep vein thrombosis subgroup (*p* = 0.23), pulmonary embolism subgroup (*p* = 1), stroke subgroup (*p* = 0.38), and myocardial infarction subgroup (*p* = 0.15) were similar in both groups. Subgroup analysis on RCTs with low risk of bias showed that TXA was associated with reduced mortality and hemorrhagic expansion. TXA was associated with reduced vascular occlusive events (RR 0.85 [0.73, 0.99], *p* = 0.04; I^2^: 4%). GRADE was performed for the RCT with low risk of bias subgroup, it showed a high certainty of evidence for lower mortality, less hemorrhage expansion, and similar need for neurosurgical intervention in TXA group compared to placebo group.

**Conclusion:**

TXA was associated with reduced mortality and hemorrhagic expansion but similar need for neurosurgical intervention and unfavorable GOS. Vascular occlusive events were slightly lower in TXA group on subgroup analysis of RCTs with low risk of bias.

## Background

The worldwide incidence of traumatic brain injury (TBI) is approximately 69 million a year [1], in which road injuries and falls contributed the most [2]. Intracranial bleeding, which is frequently associated with TBI, increases mortality [3]. Furthermore, the release of brain phospholipids and tissue factors due to TBI may precipitate coagulopathy [4]. Coagulopathy developed in around one-third of patients with severe TBI which is associated with hemorrhage expansion, poor neurological outcome, and mortality [5–7].

Tranexamic acid (TXA) can inhibit fibrinolysis by displacing plasminogen from fibrin and also inhibits enzymatic degradation by plasmin. TXA was also associated with enhanced clot strength, reduction in trauma-induced coagulopathy, and prevention of hyperfibrinolysis [8]. However, clinical trials demonstrate conflicting results regarding the benefits of the TXA in TBI patients [9–12]. The risk of thromboembolic complications is also uncertain as one study reported a significantly higher risk of pulmonary embolism (PE) in patients treated with TXA [9]. These results pose a conundrum for TBI management. This systematic review and meta-analysis aimed to synthesize the latest evidence on the efficacy and safety of TXA on TBI. This systematic review and meta-analysis adhered to the PRISMA guidelines/methodology.

## Methods

### Search strategy

**Fig. 1:**
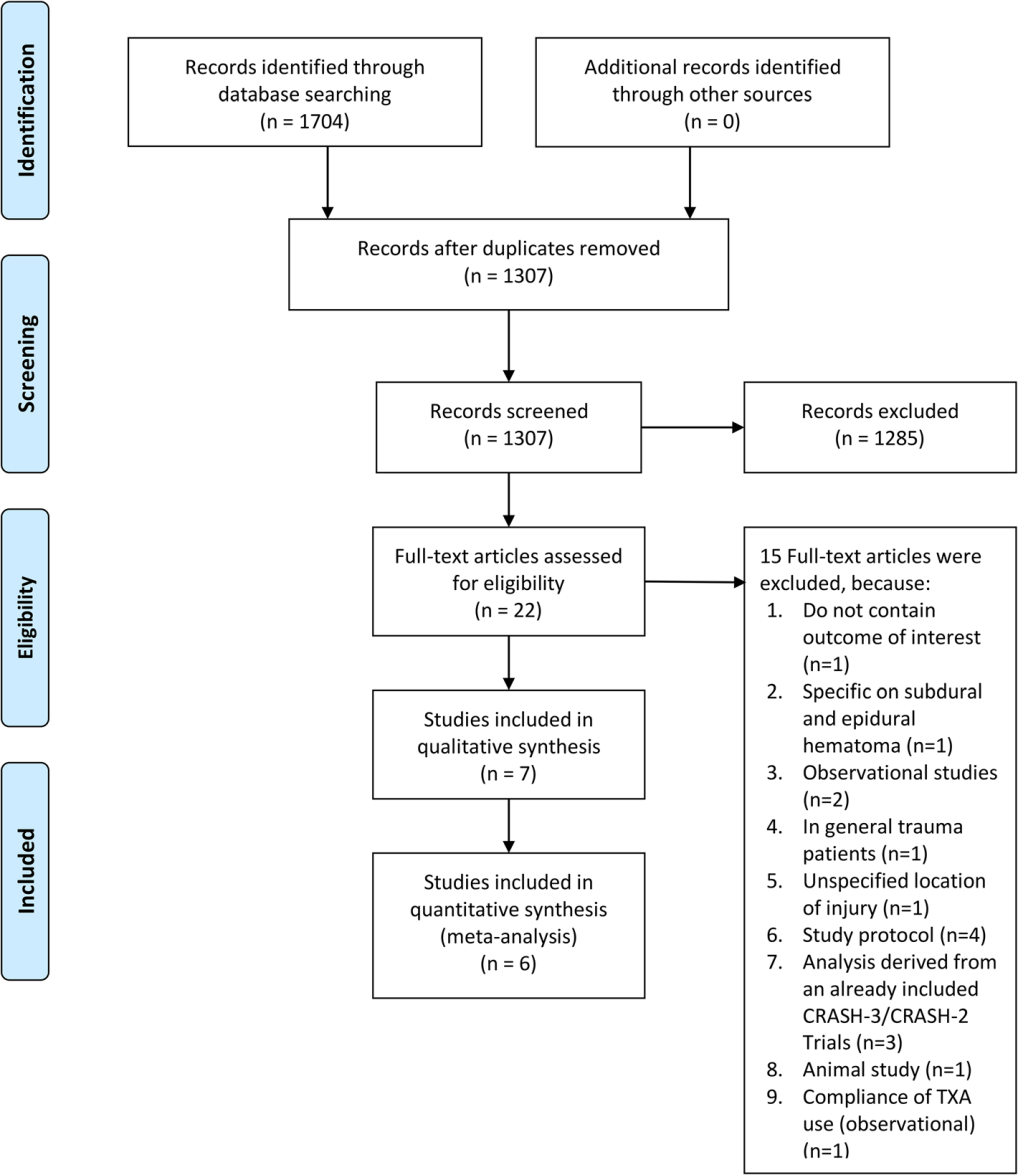
Study flow diagram

We performed a systematic literature search on topics that compared intravenous TXA to placebo in patients with TBI with keywords [“tranexamic acid” and “traumatic brain injury”] and its synonym from inception up until January 2020 through PubMed, EuropePMC, Cochrane Central Database, ScienceDirect, ProQuest, ClinicalTrials.gov, and hand-sampling from potential articles cited by other studies. The records were then systematically evaluated using inclusion and exclusion criteria. We also perform hand-sampling from references of the included studies. Two researchers (J.J and R.P) independently performed an initial search, discrepancies were resolved by discussion. A Preferred Reporting Items for Systematic Reviews and Meta-Analyses (PRISMA) flowchart of the literature search strategy of studies was presented in Fig. 1.

### Selection criteria

The inclusion criteria for this study are studies that compared intravenous TXA to placebo in patients with TBI. We include all related clinical researches/original articles and exclude animal studies, case reports, review articles, and non-English language articles.

### Data extraction

Data extraction and quality assessment were performed by two independent authors (J.J and R.P) using standardized extraction form which includes authors, year of publication, study design, sample size, subject characteristics, mortality, hemorrhagic expansion, need for neurosurgical intervention, unfavorable Glasgow Outcome Scale (GOS), deep vein thrombosis (DVT), PE, ischemic stroke, and myocardial infarction (MI).

The primary outcome was mortality, hemorrhagic expansion, need for neurosurgical intervention, and unfavorable GOS. The secondary outcomes were vascular occlusive events defined as DVT, PE, ischemic stroke, and MI.

### Statistical analysis

To perform the meta-analysis, we used RevMan version 5.3 software (Cochrane Collaboration) and STATA 16.0 (StataCorp LP). We used the risk ratio (RR) and a 95% CI as a pooled measure for dichotomous data. Inconsistency index (I^2^) test, which ranges from 0 to 100%, was used to assess heterogeneity across studies. A value above 50% or *p* < 0.10 indicates a statistically significant heterogeneity. We used the Mantel-Haenzsel method for RR, with the random-effect model regardless of heterogeneity. Small study effect was assessed using a regression-based test (Harbord test) for binary outcomes. Regression-based Egger’s test was also performed for the risk of publication bias. Cochrane Risk of Bias Assessment tool (Cochrane Collaboration) will be used to assess the risk of bias for RCTs. Subgroup analysis was performed for RCTs with a low risk of bias. All *P* values were two-tailed with a statistical significance set at 0.05 or below. The certainty of the evidence for RCTs with low risk of bias was assessed by using the Guideline Development Tool by GRADEpro GDT.

## Results

### Study selection and characteristics

We found a total of 1704 results, and 1307 records remained after the removal of duplicates. One thousand two hundred eighty-five records were excluded after screening the title/abstracts. After assessing 22 full-text for eligibility, we excluded 15 because 1) do not contain outcome of interest (*n* = 1), 2) specific on subdural and epidural hematoma (*n* = 1), 3) observational studies (*n* = 2), 4) in general trauma patients (n = 1), 5.) unspecified location of injury (n = 1), 6) study protocol (*n* = 4), 7) analysis derived from an already included CRASH-3/CRASH-2 Trials (*n* = 3), 8) animal study (n = 1), 9) compliance of TXA use (observational) (n = 1). We included 7 studies in qualitative synthesis and 6 in meta-analysis. (Fig. 1) There were a total of 30.522patients from 7 studies [9–15]. The TXA protocol was mostly 1 g TXA infused over 10 min, followed by IV infusion of 1 g over 8 h. There was 1 study that gave bolus initially and another study that gave initial dose over 30 min. Matching placebo was specified in four studies, excluding Chakroun-Walha et al. and Jokar et al. Patients were around 30–40 years old and predominantly male. Time from injury to enrolment differs across studies (Table 1).

**Table 1:** Studies included in the systematic review

Authors	Study Design	TXA Protocol	Matching Placebo	Sample Size (n)	Age (mean ± SD, years)	Male (%)	Time from Injury Eligibility (hours)	Mean Time from Injury (hours)	Funding
CRASH-32019	Double-blind RCT	1 g TXA infused over 10 min, followed by IV infusion of 1 g over 8 h	Matching Placebo (NS)	9127 (4613/4514)	41.7 ± 19.0 vs 41.9 ± 19.0	80 vs 80	Originally 8, changed to 3 h	1.9 ± 0.7	National Institute for Health Research Health Technology Assessment, JP Moulton Charitable Trust, Department of Health and Social Care, Department for International Development, Global Challenges Research Fund, Medical Research Council, and Wellcome Trust (Joint Global Health Trials scheme).
NCT01990768 (Bolus-Maintenance Group)	Double-blind RCT	1 g TXA bolus (prehospital), followed by IV infusion of 1 g over 8 h	Matching Placebo (NS)	621 (312/309)	39 (26–57) vs 36 (25–55)	73 vs 75	2	N/A	National Heart, Lung, and Blood Institute; United States Army Medical Research Acquisition Activity
Chakroun-Walha 2018	Open-label RCT	1 g TXA infused over 10 min, followed by IV infusion of 1 g over 8 h	None (no TXA)	180 (96/84)	44 ± 20 vs 39 ± 18	M/F Ratio: 11 vs 8.3	24	N/A	None
Fakharian 2017	Double-blind RCT	1 g TXA infused over 10 min, followed by IV infusion of 1 g over 8 h	Matching Placebo (NS)	149 (74/75)	42.3 ± 18.3 vs 39.3 ± 18.1	91 vs 88	8	N/A	Kashan University of Medical Sciences
Jokar 2017	Single-blind RCT	1 g TXA infused over 10 min, followed by IV infusion of 1 g over 8 h	Unobvious Placebo	80 (40/40)	35.4 ± 14.6 vs 36.2 ± 14.9	40 vs 35	2	N/A	Arak University of Medical Sciences
CRASH-22013	Double-blind RCT	1 g TXA infused over 10 min, followed by IV infusion of 1 g over 8 h	Matching Placebo (NS)	10,060/10067	34.6 ± 14.1 vs 34.5 ± 14.4	84 vs 84	8	2.9 ± 2.6	Health Technology Assessment Programme; National Institute for Health Research
Yutthakasemsunt 2013	Double-blind RCT	1 g TXA infused over 30 min, followed by IV infusion of 1 g over 8 h	Matching Placebo (Sterile Water)	238 (120/118)	34.8 ± 16.0 vs 34.1 ± 15.3	86 vs 91	8	7.1 ± 1.29	The Thailand Research Fund

*NS* Normal Saline, *RCT* Randomized Controlled Trials, *SD* Standard Deviation, *TXA* Tranexamic Acid

### Efficacy

**Fig. 2:**
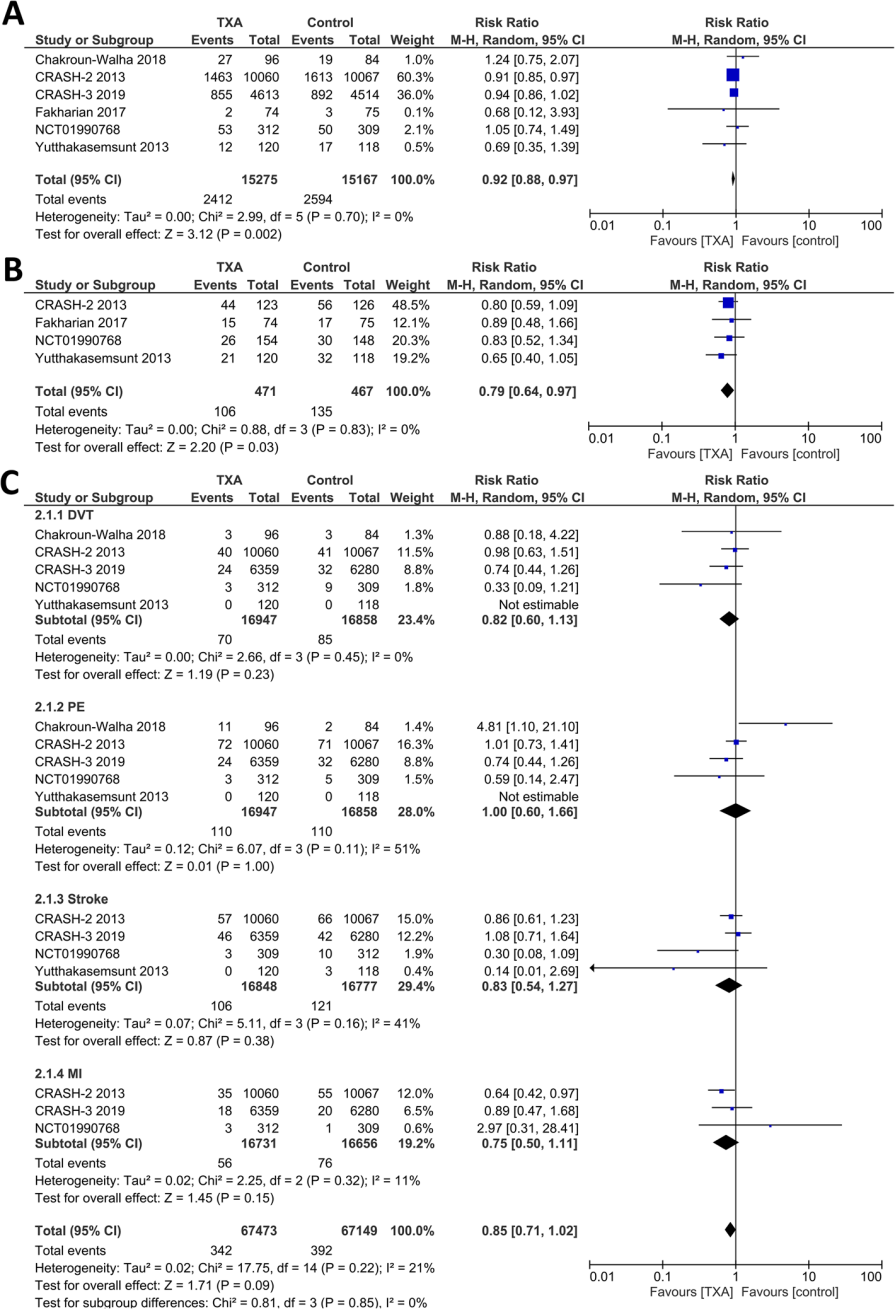
Meta-analysis for tranexamic acid versus placebo. **a** demonstrated a lower mortality rate in TXA group. **b** showed hemorrhagic expansion was less in TXA group. Vascular occlusive events (Fig. 4c), defined as DVT + PE + stroke+MI was similar in both groups. Description = DVT: Deep Vein Thrombosis; MI: Myocardial Infarction; PE: Pulmonary Embolism; TXA: Tranexamic Acid

Meta-analysis showed that TXA was associated with reduced mortality (RR 0.92 [0.88, 0.97], *p* = 0.002; I^2^: 0%, *p* = 0.70) (Fig. 2a). The rate of hemorrhagic expansion was lower in TXA group (RR 0.79 [0.64, 0.97], *p* = 0.03; I^2^: 0%, *p* = 0.83) (Fig. 2b). Both TXA and control group has a similar need for neurosurgical intervention (RR 0.99 [0.92, 1.07], *p* = 0.87; I^2^: 0%, *p* = 0.43). The unfavourable GOS on follow-up was similar in both groups (RR 0.93 [0.72, 1.21], *p* = 0.59; I^2^: 20%, *p* = 0.29). Subgroup analysis was performed for mortality in severe TBI patients, data was derived from CRASH-2 and CRASH-3 studies showed no statistically significant effect on mortality (RR 0.96 [0.91, 1.02], *p* = 0.19; I^2^: 0%, *p* = 0.43).

### Complications

The rate for vascular occlusive events were similar in both TXA and placebo groups (RR 0.85 [0.71, 1.02], *p* = 0.09; I^2^: 21%, *p* = 0.22) (Fig. 2c). The risk for DVT subgroup (RR 0.82 [0.60, 1.13], *p* = 0.23; I^2^: 0%, *p* = 0.45), PE subgroup (RR 1.00 [0.60, 1.66], *p* = 1; I^2^: 51%, *p* = 0.11), stroke subgroup (RR 0.83 [0.54, 1.27], *p* = 0.38; I^2^: 41%, *p* = 0.16), and MI subgroup (RR 0.75 [0.50, 1.11], *p* = 0.15; I^2^: 11%, *p* = 0.32) were similar in both TXA and placebo group.

### Risk of bias assessment

**Fig. 3:**
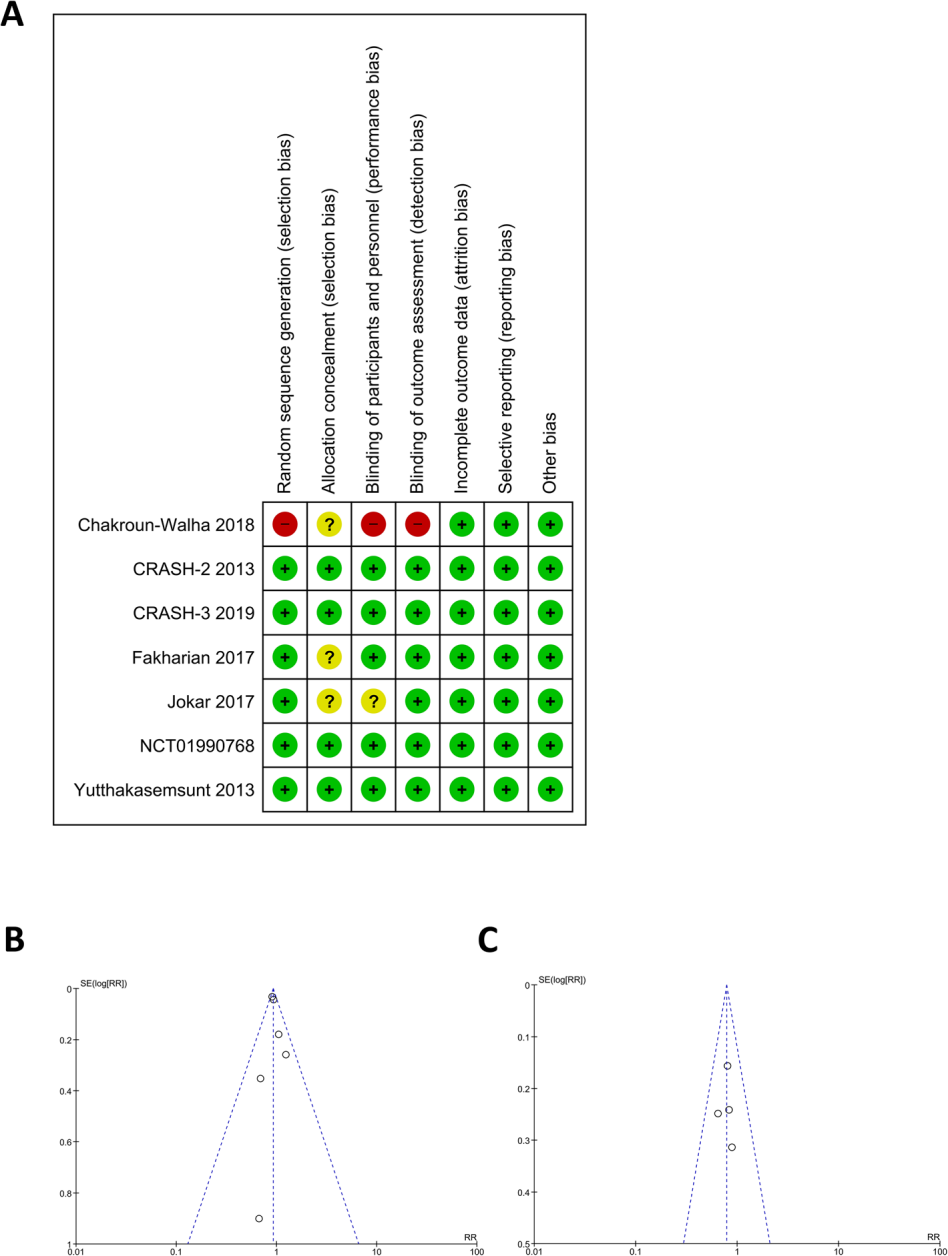
Risk of Bias Assessment. **a** showed Cochrane Risk of Bias Assessment for Randomized Controlled Trials. **b** and **c** showed funnel-plot analysis for mortality and hemorrhagic expansion respectively

Risk of bias assessment using the Cochrane risk-of-bias tool for randomized trials showed two trial (Chakroun-Walha et al. and Jokar et al.) has a high risk of bias (Fig. 3a). The remaining 5 trials have a low risk of bias. The funnel-plot analysis showed a relatively symmetrical shape for mortality (Fig. 3b) and symmetrical shape for hemorrhagic expansion (Fig. 3c). Regression-based Harbord’s test and Egger’s test were not statistically significant for all outcomes (Table 2).

**Table 2:** Summary of Meta-analysis

Outcomes	Risk Ratio (95% Confidence Interval), *p-*value	Heterogeneity (I^2^), *p*-value	Harbord’s Test	Egger’s Test	Number of Studies
Mortality	0.92 [0.88, 0.97], 0.002	0%, 0.70	0.671	0.710	6
Hemorrhagic Expansion	0.79 [0.64, 0.97], 0.03	0%, 0.83	0.623	0.751	4
Need for Neurosurgical Intervention	0.99 [0.92, 1.07], 0.87	0%, 0.43	0.332	0.347	5
Unfavourable Glasgow Outcome Scale	0.93 [0.72, 1.21], 0.59	20%, 0.29	0.106	0.136	3
Vascular Occlusive Events^a^	0.85 [0.71, 1.02], 0.09	21%, 0.22	0.513	0.82	5
DVT	0.82 [0.60, 1.13], 0.23	0%, 0.45	0.405	0.486	5
PE	1.00 [0.60, 1.66], 1	51%, 0.11	0.726	0.496	5
Stroke	0.83 [0.54, 1.27], 0.38	41%, 0.16	0.105	0.051	4
MI	0.75 [0.50, 1.11], 0.15	11%, 0.32	0.124	0.149	3
**RCT with Low Risk of Bias Subgroup**					
Mortality	0.92 [0.87, 0.97], 0.001	0%, 0.80	0.795	0.823	5
Hemorrhagic Expansion	0.79 [0.64, 0.97], 0.03	0%, 0.83	0.623	0.751	4
Need for Neurosurgical Intervention	0.99 [0.89, 1.12], 0.93	5%, 0.37	0.534	0.472	4
Unfavourable Glasgow Outcome Scale	0.93 [0.72, 1.21], 0.59	20%, 0.29	0.106	0.136	3
Vascular Occlusive Events^a^	0.85 [0.73, 0.99], 0.04	4%, 0.40	0.084	0.087	4
DVT	0.79 [0.53, 1.19], 0.26	25%, 0.27	0.170	0.392	4
PE	0.91 [0.70, 1.20], 0.52	0%, 0.51	0.383	0.542	4
Stroke	0.83 [0.54, 1.27], 0.38	41%, 0.16	0.105	0.051	4
MI	0.75 [0.50, 1.11], 0.15	11%, 0.32	0.124	0.149	3

*DVT* Deep Vein Thrombosis, *MI* Myocardial Infarction, *PE* Pulmonary Embolism

^a^indicates DVT + PE + Stroke+MI

### Subgroup analysis for randomized controlled trials with low risk of bias

**Fig. 4:**
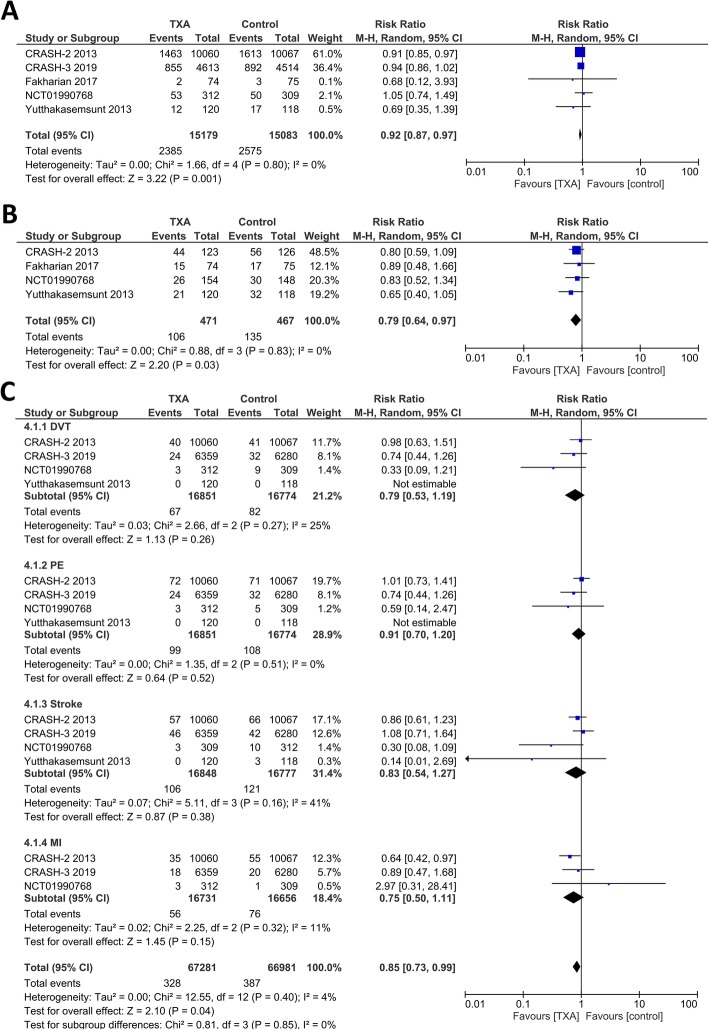
Subgroup analysis for studies with low risk of bias. **a** demonstrated a lower mortality rate in TXA group. **b** showed hemorrhagic expansion was less in TXA group. Vascular occlusive events (**c**), defined as DVT + PE + stroke+MI was lower in TXA group. Description = DVT: Deep Vein Thrombosis; MI: Myocardial Infarction; PE: Pulmonary Embolism; TXA: Tranexamic Acid

In this subgroup analysis, Chakroun-Walha et al. and Jokar et al. were excluded due to high risk of bias. Meta-analysis showed that TXA was associated with reduced mortality (RR 0.92 [0.87, 0.97], *p* = 0.001; I^2^: 0%, *p* = 0.80) (Fig. 4a). The rate of hemorrhagic expansion was lower in TXA group (RR 0.79 [0.64, 0.97], *p* = 0.03; I^2^: 0%, *p* = 0.83) (Fig. 4b). Both TXA and control group has a similar need for neurosurgical intervention (RR 0.99 [0.89, 1.12], *p* = 0.93; I^2^: 5%, *p* = 0.37). The unfavourable GOS on follow-up was similar in both groups (RR 0.93 [0.72, 1.21], *p* = 0.59; I^2^: 20%, *p* = 0.29). The rate for vascular occlusive events were lower in TXA group (RR 0.85 [0.73, 0.99], *p* = 0.04; I^2^: 4%, *p* = 0.40) (Fig. 4c). The risk for DVT subgroup (RR 0.79 [0.53, 1.19], *p* = 0.26; I^2^: 25%, *p* = 0.27), PE subgroup (RR 0.91 [0.70, 1.20], *p* = 0.52; I^2^: 0%, *p* = 0.51), stroke subgroup (RR 0.83 [0.54, 1.27], *p* = 0.38; I^2^: 41%, *p* = 0.16), and MI subgroup (RR 0.75 [0.50, 1.11], *p* = 0.15; I^2^: 11%, *p* = 0.32) were similar in both TXA and placebo group.

### GRADE approach

Grading of Recommendations Assessment, Development, and Evaluation (GRADE) were performed for the RCT with low risk of bias subgroup, it showed a high certainty of the evidence for lower mortality, less hemorrhage expansion, and a similar need for neurosurgical intervention in TXA group compared to the placebo group. The certainty of evidence was moderate for the similar unfavorable GOS, less vascular occlusive events, a similar rate of DVT, and a similar rate of MI in the TXA group compared to the placebo group. (Table 3).

**Table 3:** GRADE Approach for RCTs with low risk of bias

Certainty assessment							№ of patients		Effect		Certainty
№ of studies	Study design	Risk of bias	Inconsistency	Indirectness	Imprecision	Other considerations	Tranexamic Acid	placebo	Relative (95% CI)	Absolute (95% CI)	
Mortality											
5	randomised trials	not serious	not serious	not serious	not serious	none	2385/15179 (15.7%)	2575/15083 (17.1%)	**RR 0.92** (0.87 to 0.97)	**14 fewer per 1000** (from 22 fewer to 5 fewer)	⨁⨁⨁⨁ HIGH
Hemorrhage Expansion											
4	randomised trials	not serious	not serious	not serious	not serious	none	106/471 (22.5%)	135/467 (28.9%)	**RR 0.78** (0.63 to 0.97)	**64 fewer per 1000** from 107 fewer to 9 fewer)	⨁⨁⨁⨁ HIGH
Need for Neurosurgical Intervention											
4	randomised trials	not serious	not serious	not serious	not serious	none	1113/10566 (10.5%)	1125/10569 (10.6%)	**RR 0.99** (0.89 to 1.12)	**1 fewer per 1000** (from 12 fewer to 13 more)	⨁⨁⨁⨁ HIGH
Unfavourable GOS											
3	randomised trials	not serious	not serious	not serious	serious ^a^	none	137/455 (30.1%)	147/463 (31.7%)	**RR 0.93** (0.72 to 1.21)	**22 fewer per 1000** (from 89 fewer to 67 more)	⨁⨁⨁◯ MODERATE
Vascular Occlusive Events											
4	randomised trials	not serious	serious ^b^	not serious	not serious	none	328/67281 (0.5%)	387/66981 (0.6%)	**RR 0.85** (0.73 to 0.99)	**1 fewer per 1000** (from 2 fewer to 0 fewer)	⨁⨁⨁◯ MODERATE
Vascular Occlusive Events - DVT											
4	randomised trials	not serious	not serious	not serious	serious ^a^	none	67/16851 (0.4%)	82/16774 (0.5%)	**RR 0.79**(0.53 to 1.19)	**1 fewer per 1000** (from 2 fewer to 1 more)	⨁⨁⨁◯ MODERATE
Vascular Occlusive Events - MI											
3	randomised trials	not serious	not serious	not serious	serious ^a^	none	56/16731 (0.3%)	76/16656 (0.5%)	**RR 0.75** (0.50 to 1.11)	**1 fewer per 1000** (from 2 fewer to 1 more)	⨁⨁⨁◯ MODERATE

*CI* Confidence interval, *RR* Risk ratio, *TXA* Tranexamic Acid

**Explanations**

a. Confidence intervals included potential for important harm or benefit and the risk ratio < 0.75

b. Heterogeneity > 40%

## Discussion

This meta-analysis showed that TXA was associated with reduced mortality and hemorrhagic expansion. Vascular occlusive events were slightly lower in TXA group on a subgroup analysis of RCTs with a low risk of bias.

TXA is a trans-stereoisomer of 4-(aminomethyl)cyclohexane-carboxylic acid) binds to plasminogen via 5-lysine binding sites [16]. It prevent plasmin activation, reduces fibrinolysis, and stabilizes clot, without enhancing new clot formation [17]. Early TXA administration < 60 min has been shown to attenuate endothelial apoptosis and necrosis [18]. TXA has been shown to modulate pulmonary inflammation in trauma-induced acute lung injury [19]. In a TBI animal model, a potentially beneficial inflammatory and immune modulation were demonstrated after TXA administration [20]. Furthermore, TXA was also shown to be associated with elevated immune activation in a post-TBI pneumonia animal model [21].

Aside from the included studies, there was an observational study reported that TXA administration in patients with cerebral contusions or traumatic subarachnoid hemorrhage was independently associated with a reduced mortality rate [22]. An RCT showed that TXA was associated with reduced intraoperative bleeding but not hemoglobin change in patients with epidural, subdural, and intraparenchymal hemorrhage [23, 24].

The confidence intervals of vascular occlusive events subgroup included the potential for important harm or benefit and the risk ratio < 0.75. Hence, there is a possible benefit of TXA on the incidence of DVT, PE, stroke, and MI. Although the pooled effect estimate displayed a null-effect, the larger sample size may be required because the incidence of these events might be too low to demonstrate any significant benefits. This possibility is further strengthened that by combining all of the subgroup, the vascular occlusive events are reduced in the TXA group. This finding however, might be subjected to potential confounders. In a meta-analysis of TXA use in intertrochanteric fracture, the rate of vascular occlusive events was similar in both TXA and control groups [25]. If the benefits were proven to be true, it might be due to mechanism unrelated to anti-fibrinolysis in TBI patients, possibly due to its innate anti-inflammatory, protection against endothelial injury, and platelet improving function [16, 26]. Thromboembolic events stem from endothelial injury and inflammation [27, 28], attenuation of these factors may prove to be protective.

The timing of administration was less certain, the mean time to injury was assessed in only three studies. CRASH-3 trial showed that the mortality benefits after adjustment were most pronounced when TXA was given < 3 h in mild-moderate Glasgow Coma Scale (GCS) score, however, mortality seemed to be the same in severe GCS score [13]. Which is in accordance with the abovementioned study that the early administration is better [18]. In a small RCT by Yutthakasemsunt et al., no mortality benefit was demonstrated in a mean time from injury of 7.1 h [11]. However, CRASH-2 trial that enrolls patients up to 8 h after injury, showed the potential benefit of TXA. It should be noted that CRASH-2 trial excluded patients with isolated TBI and the benefits of TXA might be more pronounced in this trial [10]. It is possible that if the analysis was conducted on patients with mild-moderate GCS and injury < 3 h, the benefits of TXA might be more pronounced, however, subgroup analysis or meta-regression is not possible in the current meta-analysis due to lack of available data.

### Practical implications

TXA 1 g infused over 10 min, followed by IV infusion of 1 g over 8 h, may be used to reduce the risk of hemorrhage expansion and slightly reduce mortality rate. There seemed to be no thrombotic repercussions of TXA. The timing was less certain, based on CRASH-3 study, earlier administration equals better outcomes and encouraged to be given within 3 h. The benefits seemed to be demonstrable if given within 8 h, however, further research is required before making a definite conclusion. Elderly patients and patients with high thromboembolic risk were not adequately studied and a cautious multidisciplinary consideration should be weighed.

### Limitations

This systematic review and meta-analysis have several limitations; the risk of publication bias cannot be excluded even though the funnel plot was symmetrical because the number of studies was < 10. Meta-regression cannot be performed due to lack of studies, the meta-regression analysis may provide data on whether the result will be affected by covariates in the studies which are important to determine potential confounders in this study. Only a few studies reported the average time from injury to TXA administration and stratify them, hence, dose-response meta-analysis cannot be conducted. However, despite these limitations, the heterogeneity was low in the majority of analysis and the risk of bias was low in subgroup analysis providing a moderate-high certainty of evidence. The vascular occlusive events and their subgroups require further investigation, a double-blind RCT with a large sample size may demonstrate the benefits or confirm the null-effect. The research on the elderly and patients with high thromboembolic risk was also lacking.

## Conclusion

TXA was associated with reduced mortality and hemorrhagic expansion but a similar need for neurosurgical intervention and unfavorable GOS. Vascular occlusive events were slightly lower in the TXA group on a subgroup analysis of RCTs with low risk of bias, but the incidence of DVT, PE, stroke, and MI individually were similar in both TXA and control groups. Large double-blind RCT(s) is still needed to assess the potential benefit on the vascular occlusive events outcome. We also encourage researches on the elderly and patients with high thromboembolic risk.

## Data Availability

All data generated or analysed during this study are included in this published article. Corresponding author (J.J) can be contacted for more information.

## Abbreviations

DVT: Deep vein thrombosis
GCS: Glasgow coma scale
GOS: Glasgow outcome scale
MI: Myocardial infarction
PE: Pulmonary Embolism
RCT: Randomized controlled trial
TBI: Traumatic brain injury
TXA: Tranexamic acid
